# Supplementary material for: A Critical View of the Application of the APEX Software (Aqueous Photochemistry of Environmentally-Occurring Xenobiotics) to Predict Photoreaction Kinetics in Surface Freshwaters
Source: Molecules. 2019 Dec 18;25(1):9. doi: 10.3390/molecules25010009 (PMC7017383; doi:10.3390/molecules25010009)
Supplement: Supplementary file 1 [file molecules-25-00009-s001.zip › 3.2.4_gcc-4.4.0.zip!/3.2.4_gcc-4.4.0/3.2.4_gcc-4.4.0/doc/octave/html/interpreter/Global-Variables.html]

Global Variables - Untitled


Next: Persistent Variables,
Up: Variables


---

### 7.1 Global Variables

A variable that has been declared global may be accessed from
within a function body without having to pass it as a formal parameter.

A variable may be declared global using a `global` declaration
statement. The following statements are all global declarations.

```
     global a
     global a b
     global c = 2
     global d = 3 e f = 5
```

A global variable may only be initialized once in a `global`
statement. For example, after executing the following code

```
     global gvar = 1
     global gvar = 2
```

the value of the global variable `gvar` is 1, not 2. Issuing a
clear gvar command does not change the above behavior, but
clear all does.

It is necessary declare a variable as global within a function body in
order to access it. For example,

```
     global x
     function f ()
       x = 1;
     endfunction
     f ()
```

does *not* set the value of the global variable `x` to 1. In
order to change the value of the global variable `x`, you must also
declare it to be global within the function body, like this

```
     function f ()
       global x;
       x = 1;
     endfunction
```

Passing a global variable in a function parameter list will
make a local copy and not modify the global value. For example, given
the function

```
     function f (x)
       x = 0
     endfunction
```

and the definition of `x` as a global variable at the top level,

```
     global x = 13
```

the expression

```
     f (x)
```

will display the value of `x` from inside the function as 0,
but the value of `x` at the top level remains unchanged, because
the function works with a *copy* of its argument.

— Built-in Function: **isglobal** (name)  
> Return 1 if name is globally visible. Otherwise, return 0. For
> example,
>
> ```
>           global x
>           isglobal ("x")
>                => 1
> ```
